# Cisplatin-Induced Rab27A/B Exosomal PD-L1 Axis Suppresses Antitumor Immunity and Correlates with Poor 5-Year Survival in Lung Squamous Cell Carcinoma and Adenocarcinoma

**DOI:** 10.3390/cimb48070697

**Published:** 2026-07-09

**Authors:** Jing-Quan Zheng, Chin-Hua You, Tung-Yu Tiong, Bo-Jung Chen, Jou-Chun Chou

**Affiliations:** 1 Division of Pulmonary Medicine, Department of Internal Medicine, Shuang Ho Hospital, Taipei Medical University, New Taipei City 235041, Taiwan; 16044@s.tmu.edu.tw (J.-Q.Z.); 19166@s.tmu.edu.tw (C.-H.Y.); 2 Graduate Institute of Clinical Medicine, College of Medicine, Taipei Medical University, Taipei 110301, Taiwan; 3 Division of Pulmonary Medicine, Department of Internal Medicine, School of Medicine, College of Medicine, Taipei Medical University, Taipei 110301, Taiwan; 4 Division of Thoracic Surgery, Department of Surgery, Shuang Ho Hospital, Taipei Medical University, New Taipei City 235041, Taiwan; 09262@s.tmu.edu.tw; 5 Department of Pathology, Shuang Ho Hospital, Taipei Medical University, New Taipei City 235041, Taiwan; b8801061@gmail.com; 6 Department of Pathology, School of Medicine, College of Medicine, Taipei Medical University, Taipei 110301, Taiwan; 7 Department of Medical Research, Shuang Ho Hospital, Taipei Medical University, New Taipei City 235041, Taiwan

**Keywords:** cisplatin, PD-L1, exosome, RAB27A, RAB27B

## Abstract

Current research primarily focuses on post-treatment resistance, leaving the immediate impact of cisplatin on the tumor–immune interaction poorly understood. Tumor cells were co-cultured with immune cells to assess immune cell activation. The correlation with RAB27A/B and CD274 expression levels in non-small cell lung cancer (NSCLC) were analyzed using TCGA. The relationship between RAB27A/B and the tumor microenvironment was assessed by TIMER2.0. Cisplatin significantly increased PD-L1 mRNA levels and surface protein expression, detected by qPCR, flow cytometry and confocal microscopy. In the co-culture assay, cisplatin-induced cell surface PD-L1 protein levels inhibited the activation of Jukat T-cells. Cisplatin also upregulated genes associated with exosome secretion and increased total exosome particle counts and secretion per cell. Pretreatment with GW4869, an exosome release inhibitor, significantly enhanced cisplatin sensitivity in HCC827 cells. While GW4869 reduced intracellular RAB27A/B and EV PD-L1 levels, it did not alter cellular PD-L1 expression. Bioinformatic analysis further revealed that high RAB27A/B expression correlates with poor 5-year survival in NSCLC and is negatively associated with CD8+ T-cell activation, while positively correlating with cancer-associated fibroblasts. Both RAB27A and RAB27B gene expression levels positively correlated with CD274. Conclusions: RAB27A and RAB27B may be crucial proteins in modulating cisplatin-induced exosomal PD-L1 levels and the tumor microenvironment.

## 1. Introduction

Lung cancer is the leading cause of cancer-related mortality worldwide and is responsible for approximately 1.8 million deaths each year according to Global Cancer Statistics 2020 [1,2]. Histologically, lung cancer is classified into non-small cell lung cancer (NSCLC) and small cell lung cancer (SCLC), with NSCLC accounting for approximately 85% of cases [3]. With therapeutic advances in recent years, including targeted therapies (e.g., EGFR, ALK, and KRAS inhibitors), immune checkpoint inhibitors (ICIs) and chemoimmunotherapy, there are significant improvements in NSCLC prognosis [4]. While chemoimmunotherapy extends overall and progression-free survival compared to monotherapy, approximately 50% of patients still do not respond to this treatment [5].

Cisplatin, a platinum-based chemotherapeutic, has been a mainstay of lung cancer treatment for decades, particularly for patients not suitable for targeted therapy, such as EGFR or ALK inhibitors. However, over 60% of patients treated with cisplatin eventually develop resistance to the drug. In addition, cisplatin treatment often leads to side effects such as nephrotoxicity [6], neurotoxicity, nausea, bone marrow suppression (leukopenia), and hearing loss [3,4,7]. Contemporary international guidelines now recommend first-line chemoimmunotherapy for most patients with metastatic NSCLC, based on multiple phase III trials showing durable overall-survival (OS) benefits. The ASCO 2024–2025 guidelines recommend pembrolizumab + platinum/pemetrexed for non-squamous disease and pembrolizumab + platinum/taxane for squamous disease as standards of care [8].

Programmed death-ligand 1 (PD-L1) expressed on tumor cells binds to programmed death-1 (PD-1) receptors on activated T-cells, transmitting inhibitory signals that suppress T-cell proliferation, effector function, cytokine production and cytotoxicity. ty [9]. The KEYNOTE-189 randomized phase III clinical trial evaluated pembrolizumab plus platinum-pemetrexed combination therapy for advanced NSCLC. Clinical and pre-clinical data show that cisplatin can upregulate PD-L1 expression via activation of DNA damage response pathways, such as JAK/STAT and NF-κB signaling [10,11]. This upregulation may contribute to immune evasion and provide improved outcomes in chemoimmunotherapy combinations, as in the KEYNOTE-189 trial [12,13].

The biogenesis of exosomes is an intracellular dynamic endocytic process, where they are transported to the cell membrane via the RAB protein family (RAB5, 7, 11, 27A, 27B, and 35) [14]. Upon fusion with the cell membrane, they are released into the extracellular environment to form extracellular vesicles. Ultimately, these vesicles of varying diameters are collectively referred to as extracellular vesicles (EVs) [15]. Exosomes are lipid bilayer structures with diameters ranging from 30 to 150 nm [16] and are widely present in biological fluids, such as urine, saliva, blood, milk, and semen [17,18]. Rab27-related molecules lead to discrete disorders such as pigment dilution and immunodeficiency and are associated with some common diseases including diabetes and cancer. In breast cancer, melanoma, and bladder cancer tissues, the overexpression of RAB27A/B is associated with disease progression and metastasis [19]. However, studies in lung cancer remain sparse; it has been reported that RAB27A regulates exosome secretion in A549 cells [20], and RAB27B expression is negatively correlated with patient survival in squamous cell lung cancer [21]. Nevertheless, the correlations of RAB27A/B expression with cisplatin sensitivity, patient survival across distinct NSCLC subtypes, and immune infiltration remain to be fully elucidated. PD-L1 exists in multiple functional units. PD-L1 on the tumor cell surface is the primary therapeutic target of ICIs, modulating local immune suppression within the tumor microenvironment. Clinical data shows that circulating exosomal PD-L1 also holds clinical significance in many cancers, including NSCLC, melanoma, and renal cell carcinoma [22,23,24]. In patients with advanced melanoma, high expression of circulating exosomal PD-L1 is associated with the failure of ICI therapy. During the early stages of treatment, whether exosomal PD-L1 expression levels increase can be used to distinguish responders from non-responders. The expression level of exosomal PD-L1 is related to the inhibition of T-cell activation [25]. Prior to treatment, circulating exosomal PD-L1 levels below 25.96 pg/mL are associated with a better survival rate [26]. Plasma exosomal PD-L1 in patients with NSCLC is also significantly higher than that in healthy donors [27]. Moreover, exosomal PD-L1, secreted via exosomes or microvesicles, circulates and can suppress T-cell activation at distant sites, including lymph nodes, thereby reducing immune surveillance and facilitating metastatic spread [25,28]; moreover, exosomal PD-L1 can bind PD-1 on T-cells remotely, mimicking the inhibitory signal of membranous PD-L1 but independent of direct cell–cell contact [29]. These mechanistic differences suggest that exosomal PD-L1 operates as a soluble, mobile immune-checkpoint reservoir distinct from tumor-surface PD-L1 and may contribute to systemic immunosuppression and subsequent treatment resistance during chemoimmunotherapy [30].

According to KEYNOTE-189 and IMpower132, the effect of cisplatin on exosome release and exosomal PD-L1 is important in chemoimmunotherapy, but the mechanism remains unclear. The present study investigates the relationship between cisplatin exposure, exosome release, and exosomal PD-L1 expression in NSCLC cells and determines whether cisplatin-induced EV-PD-L1 contributes to reduced immunotherapy responsiveness.

## 2. Materials and Methods

### 2.1. Materials

The following primary antibodies were utilized for Western blot and imaging analyses: anti-CD63, anti-ALIX, anti-CD9, and anti-CD81 (rabbit polyclonals; iREAL Biotechnology, New Taipei City, Taiwan); rabbit anti-RAB27A and anti-RAB27B (GeneTex, Irvine, CA, USA); and mouse monoclonal anti-GAPDH (Santa Cruz Biotechnology, Inc., Santa Cruz, CA, USA). Total PD-L1 expressions were examined using an anti-PD-L1 antibody from GeneTex, whereas an Abcam-sourced anti-PD-L1 antibody (Cambridge, UK) was specifically employed for confocal microscopy. Cisplatin and GW4869 were obtained from MedChemExpress (Monmouth Junction, NJ, USA).

### 2.2. Cell Lines and Culture Conditions

Human lung cancer cell lines A549, H1299, and HCC827 were used in this study. The A549 cell line was obtained from the Bioresource Collection and Research Center (BCRC, Hsinchu, Taiwan) and maintained in Ham’s F12K medium (Gibco BRL, Life Technologies, Grand Island, NY, USA). H1299 and HCC827 cells, purchased from the American Type Culture Collection (ATCC, Manassas, VA, USA), were cultured in RPMI 1640 medium (GeneDireX, Taoyuan, Taiwan). All culture media were supplemented with 10% fetal bovine serum (FBS) (Gibco BRL), 2 mM L-glutamine, 1.5 g/L sodium bicarbonate, and 1% penicillin–streptomycin. Additionally, 25 mM HEPES was included in the RPMI 1640 medium. All cells were propagated at 37 °C in a humidified atmosphere with 5% CO_2_.

### 2.3. Exosome Isolation, Characterization, and Analysis

HCC827 cells were cultured in an RPMI 1640 medium containing 10% FBS at 37 °C for 24 h in the presence of 5% CO_2_. After the cells were washed twice with a serum-free RPMI 1640 medium, the medium was switched to an RPMI 1640 medium containing 10% EV-depleted FBS, with this incubation for 24 h. Finally, the medium was collected, then centrifuged at 300× *g* for 10 min at 4 °C to precipitate the cells. The supernatant was centrifuged at 2000× *g* for 20 min and 10,000× *g* for 30min at 4 °C to eliminate cell debris. The supernatant fraction was filtered through a 0.22 μm filter. Exosomes were collected by ultracentrifugation at 100,000× *g* for 70min at 4 °C. The exosome pellet was resuspended in 10 mL of PBS and purified by ultracentrifugation at 100,000× *g* for 70 min at 4 °C (Beckman, Indianapolis, IN, USA). The purified exosomes were resuspended in PBS for subsequent experiments including Western blot and nanoparticle tracking analysis (NTA, NS300, Malvern Panalytical, Malvern, UK). Purified exosomes were dropped onto electron microscopy grids, allowed to absorb for 10 min, and then negatively stained with 2% phosphotungstic acid for 5 min. The electron microscopy grids were observed under a transmission electron microscope (Hitachi HT7700, Tokyo, Japan).

### 2.4. Flow Cytometry

To detect cell surface PD-L1 protein, 3 × 10^5^ cells were blocked with 100 μL of PBS containing 5 μL of human TruStain FcX (Biolegend, San Diego, CA, USA) at room temperature for 20 min. Subsequently, 7.5 μL of anti-FITC-human PD-L1 antibody (#393605; Biolegend) or 7.5 μL of anti-FITC-human IgG Fc (#366915; Biolegend) was added in blocking PBS buffer to stain the cells on ice for 20 min. After these cells were washed twice with a cell staining buffer (#420201; Biolegend), they were resuspended in 700 μL of cell staining buffer containing 7 μL of 7-ADD viability staining solution on ice for 5 min. All stained cells were examined using BD FACS flow cytometry.

### 2.5. Enzyme-Linked Immunosorbent Assay

The commercial human IL-2 ELISA kit (BMS221 INST, Invitrogen, Carlsbad, CA, USA/#431807, BioLegend, San Diego, CA, USA) was used to detect IL-2 release from Jurkat T-cells. For HCC827 cell and Jurkat T-cell co-culture experiments, 2.0 × 10^3^ HCC827 cells were co-cultured with 2.0 × 10^3^ Jurkat T-cells under treatment with PMA (50 ng/mL)/PHA (1 μg/mL) for 48 h. For exosome and Jurkat T-cell co-culture experiments, 3.0 × 10^8^ HCC827 or cisplatin treated-HCC827-derived exosomes were co-cultured with 3.0 × 10^4^ Jurkat T-cells under treatment with PMA (50 ng/mL)/PHA (1 μg/mL) for 48 h. The culture media were collected in 1.5 mL Eppendorf tubes after 48 h and centrifuged at 960× *g* for 5 min to remove cells. The supernatants 100 μL were loaded to the 96-well ELISA plate and the following steps were carried out. All steps of the ELISA assay were performed in conformity with instructions. Briefly, the ELISA plate was incubated at room temperature for 3 h on a shaker. After washing, the ELISA plate was incubated with 100 μL TMB Substrate Solution at room temperature for 10 min. The reaction was stopped by the addition of 100 μL per one well of Stop Solution into the ELISA plate and the absorbance was measured by a microplate reader (BioTek, Winooski, VT, USA) at 450 nm.

### 2.6. Quantitative Reverse-Transcription PCR

To measure the level of PD-L1 mRNA in HCC827 and H1299 cells, RNA in these cell lines was extracted by a commercial kit (ZYMO research, Irvine, CA, USA) after incubation for 48 h. RNA was reverse-transcribed to complementary DNA (cDNA) using the iScript cDNA synthesis kit (BioRad, Hercules, CA, USA). The mRNA levels were analyzed by SYBR Green PCR Master Mix (Thermo Fisher Scientific, South San Francisco, CA, USA) in a StepOnePlus™ System (Thermo Fisher Scientific). The mRNA level analysis was calculated by the delta-Ct value gained from the target gene Ct value minus the control gene Ct value. The mRNA level of GAPDH was used as a reference control. The primer sequences were displayed as follows: PD-L1 forward 5′-GGACAAGCAGTGACCATCAAG-3′ and reverse 5′-CCCAGAATTACCAAGTGAGTCCT-3′; GAPDH forward 5′-TGGGCTACACTGAGCACCAG-3′ and reverse 5′-CAGCGTCAAAGGTGGAGGAG-3′.

### 2.7. Immunofluorescence Staining

HCC827 cells were seeded in 24-well insert coverslips and were incubated for 24 h. Cells adhered on coverslips were fixed with 4% paraformaldehyde in PBS for 10 min, after which cells were washed with PBS and blocked with 10% BSA in PBS at room temperature for 1 h. Cells were washed three times with PBS, then incubated with primary antibodies, including anti-PD-L1 (1:500; ab213524, abcam, Cambridge, UK), at 4 °C overnight. After removing primary antibodies, cells were washed three times with PBS. Cells were incubated with secondary antibodies at room temperature for 1 h. Finally, cells were washed in PBS and mounted in mounting solution. The protein expression in cells was observed through a confocal fluorescence microscope (LEICA SP5, Wetzlar, Germany).

### 2.8. Statistical Analysis

All results are shown as mean + SEM from three to four independent experiments. The *p*-values were calculated using a Student’s two-tailed *t* test, where *p*-value < 0.05 was defined as statistically significant. Survival curves were constructed via the Kaplan–Meier method and analyzed by the log-rank test. Data was analyzed by GraphPad Prism 5.0 software.

## 3. Results

### 3.1. Cytotoxicity of Cisplatin in NSCLC Cells

**Figure 1:**
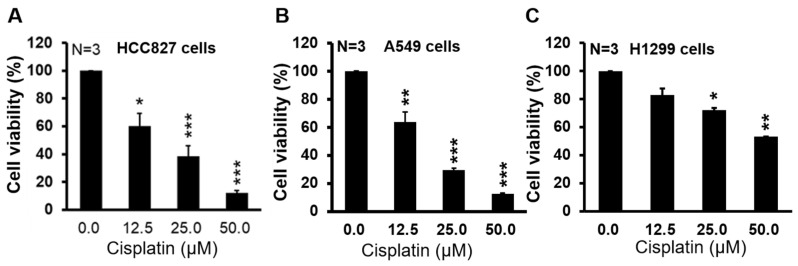
Proliferation in NSCLC cells was inhibited after treatment with cisplatin. (**A**) HCC827, (**B**) A549 and (**C**) H1299 cells were seeded in 96-well cell culture plates with incubation for 24 h and then treated with or without cisplatin with indicated concentrations for 24 h. Cell viability was measured by CCK8 kit. * *p* < 0.05, ** *p* < 0.01, and *** *p* < 0.001 vs. cisplatin 0 μM.

To investigate the effect of cell proliferation on cisplatin, we showed the viability of NSCLC cell lines including HCC827, A549 and H1299 (Figure 1A–C). The cell viability was significantly decreased after administration of cisplatin at a concentration from 12.5 to 50 μM. The values of 24 h IC50 were 16.99 ± 1.60, 16.44 ± 1.93 and 61.27 ± 0.96 μM in HCC827, A549 and H1299 cell lines, respectively (Table 1). The result showed higher sensitivity of cytotoxicity in low-metastasis cell lines, such as A549 and HCC827, than in the metastasis cell line (H1299) [31,32].

### 3.2. Association of RAB27A/B Expression with the Immune Microenvironment and Cisplatin Resistance in NSCLC Subtypes

**Figure 2:**
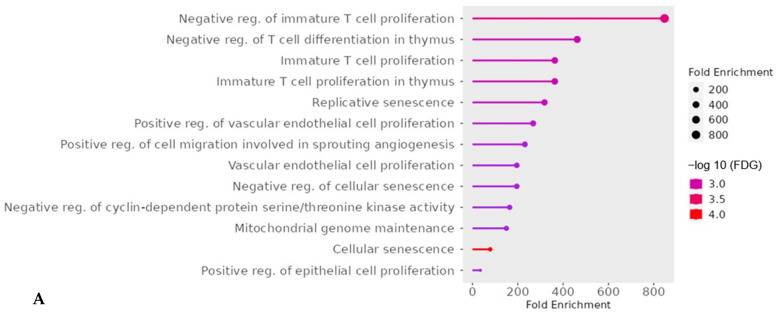

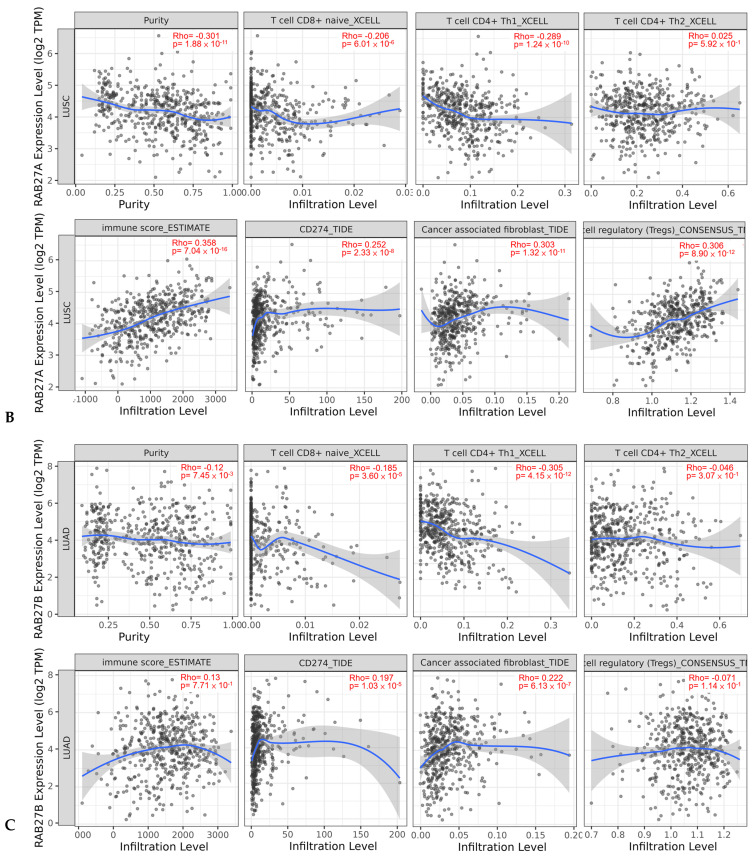
The levels of RAB27A and RAB27B are negatively correlated with the antitumor immune effect. (**A**) Enrichment analysis of top 50 differentially expressed genes associated with cisplatin resistance. (**B**) Correlation between RAB27A expression and immune microenvironment features in LUSC. (**C**) Correlation between RAB27B expression and immune microenvironment features in LUAD. Spearman correlation analysis was performed to evaluate the relationship between RAB27A/B mRNA expression levels (log2 TPM) and various TIME parameters in LUSC/LUAD. Top panels show the correlation of RAB27A/B with tumor purity and infiltration levels of naive CD8^+^ T-cells, CD4^+^ Th1 cells, and CD4^+^ Th2 cells. Bottom panels illustrate the association of RAB27A expression with immune scores (ESTIMATE), CD274 (PD-L1) expression, cancer-associated fibroblasts (CAFs), and regulatory T-cells (Tregs). Spearman’s rho and *p*-values are indicated in each plot. (**B**,**C**) The blue solid line represents the smoothed regression curve, and the grey shaded area indicates the 95% confidence interval. TIDE: Tumor Immune Dysfunction Exclusion.

To initially explore the molecular mechanisms and biological pathways underlying platinum resistance in NSCLC, we performed functional enrichment analysis on a dataset of cisplatin resistance-associated genes (Figure 2A). Strikingly, the results demonstrated that these resistance-associated genes were predominantly enriched in pathways critical to immune evasion, cell cycle regulation, and survival. Notably, negative regulation of immature T-cell proliferation exhibited the highest fold enrichment, followed by negative regulation of T-cell differentiation in thymus and cellular senescence. These findings strongly suggest that the development of cisplatin resistance in lung cancer is intimately linked to the suppression of T-cell-mediated antitumor immunity (Figure 2A). Given that T-cell suppression emerged as a prominent feature of cisplatin resistance, we next investigated whether Rab27-related molecules—known regulators of exosome-mediated immune communication—are associated with the immune microenvironment across different non-small cell lung cancer (NSCLC) subtypes. In lung squamous cell carcinoma (LUSC) tissues, RAB27A expression exhibited significant correlations with immune cell infiltration and microenvironment scores (Figure 2B). Specifically, RAB27A expression was negatively correlated with tumor purity (Rho = −0.301; *p* < 0.001), CD8^+^ T-cells and CD4^+^ Th1 cells, but demonstrated strong positive correlations with immune score (Rho = 0.358; *p* < 0.001), regulatory T-cells (Tregs) (Rho = 0.306; *p* < 0.001), cancer-associated fibroblasts (Rho = 0.303; *p* < 0.001) and crucially, the immune checkpoint molecule CD274 (PD-L1) (Rho = 0.252; *p* < 0.001) (Figure 2B). Similarly, in lung adenocarcinoma (LUAD) tissues, RAB27B expression also displayed distinct correlations with the immune landscape. RAB27B was negatively correlated with tumor purity (Rho = −0.12; *p* < 0.01), CD8^+^ T-cells, and CD4^+^ Th1 cells (Rho = −0.305; *p* < 0.001), while positively correlating with CD274 (PD-L1) expression (Rho = 0.197; *p* < 0.001), and cancer-associated fibroblasts (CAFs) (Rho = 0.222; *p* < 0.001) (Figure 2C). Taken together, these bioinformatic data first reveal that cisplatin resistance genes are heavily involved in hindering T-cell proliferation. Concurrently, the tight correlations of RAB27A/B with T-cell infiltration and PD-L1 expression imply that RAB27A/B may serve as a critical bridge modulating the tumor–immune microenvironment and driving resistance in NSCLC.

### 3.3. High RAB27A/B Expression Correlates with Poor 5-Year Survival and CD274 Expression in NSCLC

**Figure 3:**
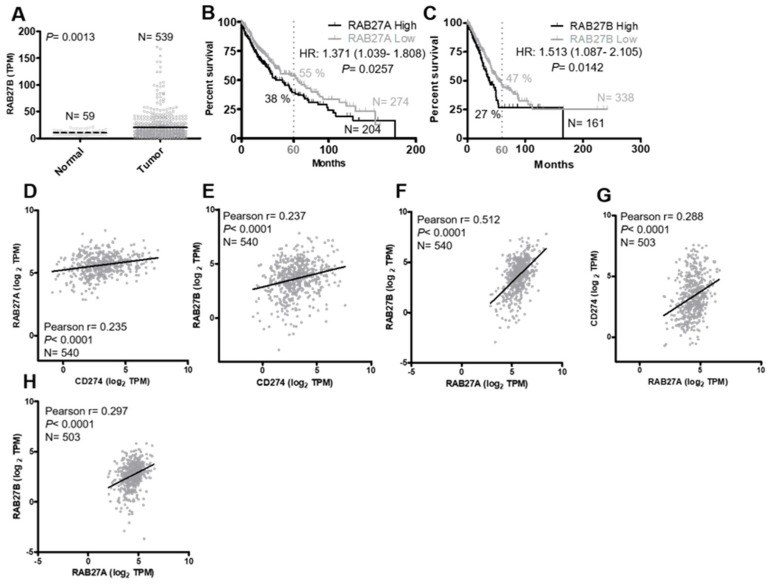
Bioinformatic analysis from the TCGA dataset. (**A**) Comparison of RAB27B mRNA expression levels between normal and tumor tissues in lung adenocarcinoma (LUAD). (**B**,**C**) Kaplan–Meier survival curves showing the overall survival of patients (LUAD and LUSC) with high vs. low expression of (**C**) RAB27B and (**B**) RAB27A. (**D**,**E**) Pearson correlation analysis between (**D**) RAB27A or (**E**) RAB27B expression levels and CD274 (PD–L1) mRNA levels in LUAD. (**F**) Pearson correlation analysis between RAB27A and RAB27B expression levels in LUAD. (**G**,**H**) Pearson correlation analysis between (**G**) RAB27A or (**H**) RAB27B expression levels and CD274 (PD–L1) mRNA levels in LUSC. (**D**–**H**) Each gray dot represents an individual patient sample, showing the gene expression levels (log_2_ TPM) on the corresponding axes. The solid black line indicates the linear regression line, representing the overall correlation trend between the two analyzed genes.

The gene expression of RAB27B is significantly higher in tumor tissues than in normal tissues in patients with lung adenocarcinoma (LUAD), with a fold change of 2.0 and a *p*-value of less than 0.01 (Figure 3A). Therefore, we further analyzed the correlation between RAB27 levels and survival rates in patients with NSCLC from the TCGA database. The results showed that the higher RAB27A and RAB27B gene expressions were associated with significantly lower survival rates in patients with NSCLC (log-rank test; *p* = 0.0257 and 0.0142; hazard ratios: 1.371 and 1.513, respectively) (Figure 3B,C). The 5-year survival rate in patients with high RAB27A expression was 38%, which is lower than that in patients with low RAB27A expression (55%) in lung squamous cell carcinoma (LUSC) tumor tissues (Figure 3B). The values of median survival in patients with high or low RAB27A expressions were 40 and 64 months, respectively (Figure 3B). The five-year survival rate in patients with high RAB27B was 27%, which is lower than that in patients with low RAB27B (47%) in LUAD tissues. The values of median survival in patients with high or low RAB27B expressions were 39 and 55 months, respectively (Figure 3C). It is well established that high exosome secretion and high PD-L1 expression are associated with poor prognosis [33,34]. Therefore, we also analyzed the correlation between CD274, RAB27A and RAB27B in LUAD from the TCGA database. The results showed that these three genes were mutually positively related to each other, with Pearson r values of 0.235, 0.237, and 0.512 and *p*-values of less than 0.0001 (Figure 3D,F). We also found a similar tendency in LUSC. The gene levels of CD274 and RAB27B were positively related to RAB27A, with *p*-values of less than 0.0001 and Pearson r values of 0.288 and 0.297 (Figure 3G,H). These results pointed out that RAB27A and RAB27B might be crucial markers to predict survival rates and determine suitability for immune checkpoint inhibitor therapy.

### 3.4. Cisplatin Treatment Enhances Cellular and Exosomal PD-L1 Levels

**Figure 4:**
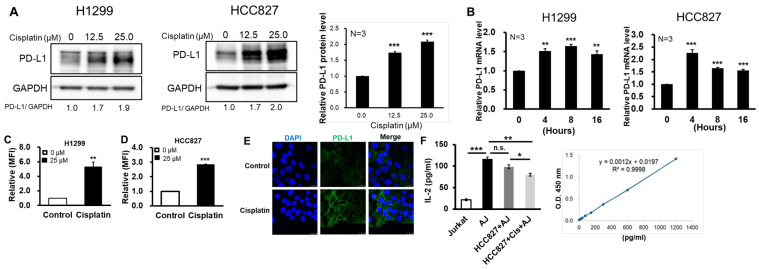
The expression levels of PD-L1 after treatment with cisplatin. (**A**) H1299 and HCC827 cells treated with or without cisplatin for 24 h. The protein expression of PD-L1 was analyzed by immunoblotting. The relative protein expression of PD-L1 in HCC827 cells is represented as a histogram (right panel). (**B**) qRT-PCR analysis of CD274. (**C**,**D**) The cell-surface PD-L1 levels were detected by flow cytometry. The relative mean fluorescence intensity (MFI) is presented as a histogram. *N* = 3. (**E**) The cell-surface PD-L1 levels were detected by confocal microscopy. (**F**) HCC827 cells co-cultured with activated Jurkat T-cell (AJ) combination with or without cisplatin (12.5 μM) for 48 h at an effector/target cell ratio = 1:1. The IL-2 release from Jurkat T-cells was measured by an ELISA assay. *N* = 3; * *p* < 0.05, ** *p* < 0.01, and *** *p* < 0.001 vs. Control.

Based on KEGG and Shiny GO analysis, platinum resistance was associated with negative regulation of T-cell proliferation (Figure 2). We investigated whether cisplatin affected PD-L1 expression in NSCLC cells. The protein level of PD-L1 increased after treatment with cisplatin with concentrations of 12.5 μM and 25 μM for 24 h in H1299 and HCC827 cells (Figure 4A). We next examined whether the cisplatin-induced upregulation of PD-L1 protein was mediated through enhanced transcription of CD274. The results revealed that PD-L1 mRNA expressions increased after administration with cisplatin (12.5 μM) at 4, 8 and 16 h (Figure 4B). To further confirm the cell surface PD-L1 level, we added cisplatin to the culture medium and collected cells to measure the intensity of fluorescence by flow cytometry. The results showed that values of MFI were significantly enhanced in cisplatin-treated cells (Figure 4C,D). These results suggested that cisplatin evaluated the PD-L1 level on the cell surface. We also observed the location of PD-L1 in HCC827 cells with or without cisplatin. The fluorescent intensity of PD-L1 was higher in cisplatin-treated cells than in the control group. The cell-surface PD-L1 level was also increased after treatment with cisplatin (Figure 4E). Tumor-surface PD-L1 has been known to downregulate T-cell activity. Here, we investigated the activity of Jurkat T-cell co-culture with HCC827 cells by measuring the concentration of IL-2 in culture medium. IL-2 release increased after treatment with PMA/PHA (Jurkat T-cell stimulatory reagents). IL-2 release was slightly decreased in the Jurkat T-cell co-culture with the HCC827 cell group but significantly decreased in that with the combination treatment with cisplatin. These results suggested that cisplatin-induced PD-L1 on the HCC827 surface could interfere with the activity in Jurkat T-cells (Figure 4F).

### 3.5. Cisplatin Promotes Exosome Release and Exosomal PD-L1

**Figure 5:**
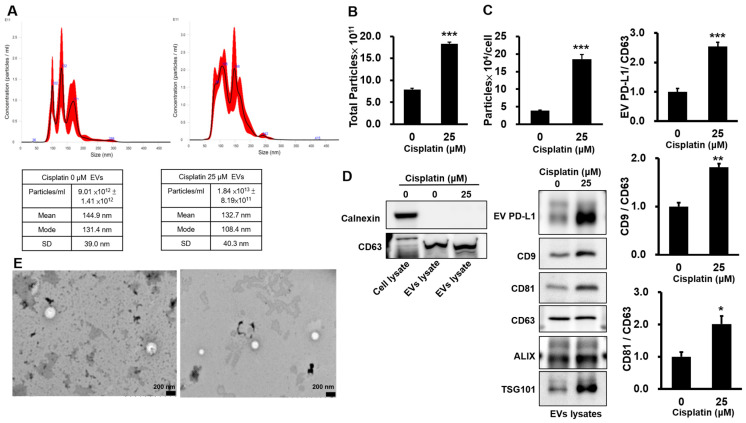
Exosome secretion was promoted in cisplatin-treated cells. (**A**) Size, number and distribution analysis of HCC827-derived exosomes by NTA. The solid line represents the mean size distribution, and the red shaded area indicates the standard deviation of the measurements. (**B**) The quantification of total particles in control and cisplatin-treated cells. (**C**) The quantification of particle numbers secreted from each cell. (**D**) Western blot analysis of exosome marker protein expression in exosome lysates. *N* = 3. (**E**) Transmission electron microscopy (TEM) image of exosomes isolated from culture medium after purification by differential centrifugation. * *p* < 0.05, ** *p* < 0.01, and *** *p* < 0.001 vs. Control.

We examined the effect of cisplatin on exosome secretion. The size and number of particles were measured by NTA (Figure 5A). We found that the number of total particles (Figure 5B) and the number of particles secreted per cell (Figure 5C) increased in cisplatin-treated cells. Exosomes were isolated by differential centrifugation and imaged using a transmission electron microscope to observe exosome morphology. The images showed that exosomes had a membrane bilayer structure and size of diameter, with diameters consistent with the NTA results (Figure 5E). The protein levels of exosome-related markers such as CD9, CD81, CD 63, TSG101 and ALIX exosome lysates were detected by Western blot. The CD63 level was used as internal control. The protein expressions of exosomal PD-L1, CD9, CD81 and TSG101 were significantly increased in cisplatin-treated cell release exosomes as compared with that in control exosomes (Figure 5D). The quantity results of CD9, CD81, TSG101 and exosomal PD-L1 increased approximately two-fold in the cisplatin-treated group (Figure 5E). These results suggested that cisplatin treatment stimulated HCC827 cells to release exosomes and enhanced the protein expression of exosomal PD-L1.

### 3.6. The Inhibitory Effect of Cisplatin on Cell Proliferation Was Increased by Exosome Inhibition

**Figure 6:**
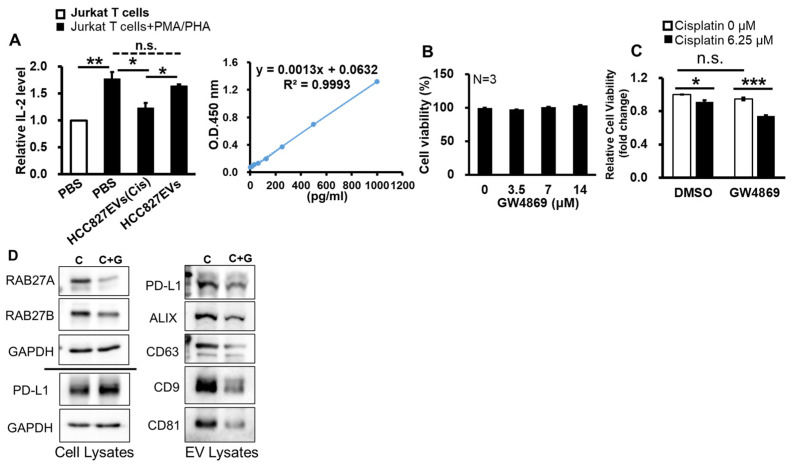
Inhibiting the release of exosomes sensitizes cells to cisplatin. (**A**) HCC827-derived exosomes co-cultured with activated Jurkat T-cells (AJ) for 48 h. The IL-2 release from Jurkat T-cells was measured by an ELISA assay. *N* = 3. (**B**) HCC827 cells were seeded in 96-well cell culture plates with incubation for 24 h and then treated with or without GW4869 with indicated concentrations for 24 h. Cell viability was measured by a CCK8 kit. (**C**) HCC827 cells were seeded in 96-well cell culture plates with incubation for 24h and then treated with or without GW4869 (10 µM) combination with or without cisplatin for 24 h. Cell viability was measured by a CCK8 kit. (**D**) The protein expressions of exosome-related markers in HCC827 cells. C: Cisplatin 6.25 µM; G: GW4869 10 µM. * *p* < 0.05, ** *p* < 0.01, and *** *p* < 0.001 vs. Control.

The above results showed that cisplatin altered exosome release in HCC827 cells by modulating exosome-formation-related proteins (Figure 5). We further investigate whether the effect of cell proliferation on cisplatin treatment was decreased after the inhibition of exosome release. IL-2 release was slightly decreased in the Jurkat T-cell co-culture with HCC827-derived exosomes but significantly decreased in cells co-cultured with exosomes from cisplatin-treated HCC827 cells (Figure 6A). These results suggested that cisplatin-induced exosomal PD-L1 could interfere with the activity in Jurkat T-cells. The exosome release antagonist GW4869 was added to HCC827 cells at a series of doses from 0 μM to 14 μM for 24 h. Cell viability was determined by a CCK-8 assay kit. The cell viability was not altered after treatment with GW4869 for 24 h (Figure 6B). This experiment confirmed that GW4869 did not induce cytotoxicity at concentrations up to 14 μM. Next, we investigated whether combining GW4869 with cisplatin led to a higher inhibition rate of cell viability. The cell viability inhibition rate was 9% in HCC827 cells treated with cisplatin alone and 22% in cells treated with GW4869 combined with cisplatin (Figure 6C). The results suggested that exosome inhibition might enhance antitumor sensitivity to HCC827 cells. We also observed the protein expression of PD-L1, RAB27 and exosome-related markers including CD63, CD9, CD81 and ALIX in cell lysates (Figure 6D, right panel) or exosome lysates (Figure 6D, right panel). In cell lysates, the protein expression of RAB27A and RAB27B decreased in cells treated with GW4869 combined with cisplatin compared with cisplatin alone. PD-L1 levels did not change significantly whether or not cells were treated with GW4869 (Figure 6D, left panel). In exosome lysates, the protein expression of CD63, CD9, CD81, ALIX and EV-PDL1 decreased in the GW4869-plus-cisplatin group compared with the cisplatin-alone group (Figure 6D, right panel). These results suggested that the inhibition of exosome secretion and exosomal PD-L1 enhanced the inhibitory effect of cisplatin on cell proliferation in HCC827 cells. RAB27A and RAB27B have been known to regulate exosome secretion.

## 4. Discussion

In the present study, we demonstrated that cisplatin treatment increased the protein expression of PD-L1 and exosomal PD-L1. Cisplatin also induced total exosome secretion and the capacity for exosome secretion per cancer cell, indicating that cisplatin maintained or increased total exosome output despite inducing apoptosis. The RAB protein family is involved in the process of endosome biogenesis. Specifically, RAB27A and RAB27B regulate the key step of trafficking endosomes to the cell membrane for secretion into the extracellular space. High levels of RAB27A and RAB27B negatively affected cytotoxic T-cell activation and reduced survival rates in LUSC and LUAD, respectively. A limitation of our result is that the Jurkat T-cell assay cannot fully replicate primary human T-cell responses, and PD-L1 blocking experiments were not performed. Although clinical dataset analysis (Figure 2B,C) strongly supports this immune suppression mechanism, further validation using primary T-cells and animal models is required in future studies.

Chen et al. generated cisplatin-resistant A549 and H69 cell lines through long-term drug selection, demonstrating that these resistant cells exhibit a marked upregulation of PD-L1 at both the protein and mRNA levels [35]. This observation is consistent with our results in Figure 2A, which revealed that platinum resistance-induced genomic alterations in NSCLC patients are strongly associated with suppressed T-cell activation and proliferation. Notably, the current first-line standard of care for advanced NSCLC involves chemoimmunotherapy regimens, such as KEYNOTE-189 or IMpower132, which concurrently administer cisplatin/carboplatin, pemetrexed, and ICIs [5]. In this context, our study distinctively differs from the aforementioned study by elucidating the acute impact of cisplatin on the immediate expression of both cellular and exosomal PD-L1—a crucial determinant of ICI therapeutic efficacy. Numerous studies have reported that the upregulation of intracellular PD-L1 in cisplatin-resistant lung cancer cells is primarily mediated by microRNAs, such as miR-181a and miR-100-5p [35,36]. In contrast, our findings demonstrate that cisplatin directly enhances the transcription of PD-L1, subsequently increasing both intracellular protein expression and exosomal PD-L1 levels. Accumulating evidence suggests that the distribution of a specific protein between intracellular compartments and the extracellular space exists in a dynamic equilibrium; a higher rate of extracellular trafficking typically leads to a corresponding decrease in the intracellular pool. To investigate this dynamic translocation and determine whether the elevated exosomal PD-L1 is driven by augmented secretion or overall protein upregulation, we used exosome secretion inhibitors (GW4869) in our experimental model.

Research on the small-molecule drug GW4869 has predominantly focused on breast and prostate cancers, leaving its role in lung cancer largely unexplored. Specifically, only two studies have evaluated the combination of GW4869 and cisplatin in lung cancer [37,38], primarily targeting exosomes secreted by cisplatin-resistant cells. To address this paucity of research, we present evidence that the combined treatment of cisplatin and GW4869 enhances the sensitivity of tumor cells to cisplatin and reduces the expression of exosomal PD-L1 induced by cisplatin. Our study also identifies a potential therapeutic vulnerability within this immune escape axis. Pharmacologic inhibition of exosome secretion using GW4869 significantly reduced the release of PD-L1-enriched exosomes, providing functional evidence that vesicle biogenesis and trafficking are critical mediators of cisplatin-induced immune modulation. These observations are consistent with prior work demonstrating that blockade of neutral sphingomyelinase-dependent exosome formation can alter vesicle release and tumor–immune interactions [39,40].

In our cohort, elevated expression of RAB27A and RAB27B—key mediators of exosome docking and secretion [41]—was significantly associated with inferior overall survival in patients with NSCLC subtypes. These findings align with emerging clinical evidence demonstrating that dynamic increases in circulating exosome-associated PD-L1 during treatment correlate with disease progression and unfavorable outcomes, particularly in patients receiving ICIs [42]. Although direct clinical response data for ICIs were not evaluated in our cohort, these results suggest that RAB27A/B may serve as potential indicators of poor prognosis and warrant further validation as predictive biomarkers for immunotherapy. While existing research on cisplatin and exosomes has primarily focused on which circular RNAs are encapsulated within exosomes derived from drug-resistant NSCLC [35,36,43], our study investigated how cisplatin alters exosome secretion. This alteration subsequently influences the tumor microenvironment, ultimately reducing the therapeutic efficiency of cisplatin and ICIs. We found that elevated levels of RAB27A and RAB27B led to increased CAF activation and reduced cytotoxicity of CD8^+^ T-cells. This phenomenon suggests that CAFs establish physical or functional barriers that prevent immune cells from infiltrating the tumor core, thereby explaining why high immune infiltration does not correlate with cytotoxic T-cell activation. The detrimental impact of RAB27A and RAB27B on the tumor microenvironment—which impairs immune-mediated tumor killing—is further reflected in patient survival outcomes. Our analysis reveals that high expression of RAB27A and RAB27B exerts a distinct adverse effect on the survival of patients with LUSC and LUAD, respectively. To date, most investigations into RAB27A/B have been conducted directly on general NSCLC [21], leaving a gap in subtype-specific analysis. Our results address this by demonstrating that the two RAB27 isoforms distinctly correspond to different subtypes of NSCLC. Koh and Song reported that Rab27A and Rab27B expression was associated with patient gender and histologic type but not with tumor node metastasis stage. Kaplan–Meier analysis revealed a poorer prognosis in LUSC patients with high Rab27B expression compared to patients with low Rab27B expression. These discrepancies between our findings and previous reports may stem from variations in sample sizes as well as the specific methodologies used for protein or gene detection [21]. From a clinical translation perspective, detecting exosomal components from peripheral blood presents technical challenges and high costs in routine oncology practice. In contrast, evaluating tumor-intrinsic RAB27 expression via standard IHC on biopsy tissues offers a highly feasible, cost-effective, and reproducible approach. Therefore, RAB27 holds significant potential as a tissue-based surrogate biomarker to predict the secretion of immunosuppressive exosomal PD-L1 following cisplatin treatment.

Our findings confirm that GW4869 downregulates RAB27A and RAB27B protein expressions and abrogates cisplatin-induced exosomal PD-L1 elevation; notably, intracellular PD-L1 levels remain unaffected, creating a tumor microenvironment conducive to ICI therapy. Nevertheless, how GW4869 modulates intracellular PD-L1 dynamics warrants further validation. It remains unclear whether the steady-state intracellular PD-L1 levels are due to inhibited exosome release causing intracellular retention, or transcriptional suppression maintaining a balance. Investigating PD-L1 mRNA alterations and cell-surface PD-L1 abundance post-GW4869 treatment will elucidate the precise mechanisms underlying the synergy between GW4869 and ICIs. We observed a downregulation of intracellular RAB27A/B following GW4869 treatment. This might be attributed to an intracellular retrograde feedback mechanism, where the pharmacological blockade of exosome budding by nSMase2 inhibition triggers a compensatory suppression of upstream Rab GTPases to maintain vesicular homeostasis, a phenomenon that warrants further investigation. Given that RAB27A and RAB27B expressions modulate key tumor microenvironment components—including CAFs and cytotoxic T-cells—our in vitro findings show that cisplatin-induced exosomes impair Jurkat T-cell activation. However, to emphasize potential clinical administration and verify whether GW4869 pretreatment synergistically improves chemoimmunotherapy efficacy, in vivo animal studies are indispensable to address the following: first, how the inhibition of exosome release by GW4869 affects CAF activation; second, whether the combination of GW4869 with chemoimmunotherapy yields a more potent suppression of tumor progression than chemoimmunotherapy alone; and third, what alterations GW4869 induces in peripheral blood immune cell composition.

## 5. Conclusions

Our study establishes exosomal PD-L1 dissemination as a previously underappreciated mechanism of chemotherapy-induced immune escape in NSCLC. This pathway likely contributes to acute cisplatin-effects during platinum-based treatment and may partially explain the heterogeneous and often transient responses observed with chemoimmunotherapy combinations. Beyond its mechanistic implications, this work provides a strong rationale for the development of exosomal PD-L1 as a real-time liquid biopsy biomarker and highlights exosome biogenesis as a promising therapeutic target to enhance the efficacy and durability of combined chemoimmunotherapy strategies.

## Figures and Tables

**Table 1:** IC_50_ concentrations calculated in A549, HCC827 and H1299 cells.

Cell Line	Ciplatin IC50 (μM)
A549	16.44 ± 1.93
HCC827	16.99 ± 1.60
H1299	61.27 ± 0.96

## Data Availability

The original contributions presented in this study are included in the article. Further inquiries can be directed to the corresponding author.

## References

[1] Sung H., Ferlay J., Siegel R.L., Laversanne M., Soerjomataram I., Jemal A., Bray F. (2021). Global Cancer Statistics 2020: GLOBOCAN Estimates of Incidence and Mortality Worldwide for 36 Cancers in 185 Countries. CA Cancer J. Clin..

[2] Siegel R.L., Miller K.D., Wagle N.S., Jemal A. (2023). Cancer statistics, 2023. CA Cancer J. Clin..

[3] Travis W.D., Brambilla E., Nicholson A.G., Yatabe Y., Austin J.H.M., Beasley M.B., Chirieac L.R., Dacic S., Duhig E., Flieder D.B. (2015). The 2015 World Health Organization Classification of Lung Tumors: Impact of Genetic, Clinical and Radiologic Advances Since the 2004 Classification. J. Thorac. Oncol..

[4] Hirsch F.R., Scagliotti G.V., Mulshine J.L., Kwon R., Curran W.J., Wu Y.L., Paz-Ares L. (2017). Lung cancer: Current therapies and new targeted treatments. Lancet.

[5] Reck M., Remon J., Hellmann M.D. (2022). First-Line Immunotherapy for Non-Small-Cell Lung Cancer. J. Clin. Oncol..

[6] Gwon M.G., Park M.H., Leem J. (2026). Magnolol Ameliorates Cisplatin-Induced Acute Kidney Injury with Activation of Nrf2-Associated Antioxidant Responses. Curr. Issues Mol. Biol..

[7] Dasari S., Tchounwou P.B. (2014). Cisplatin in cancer therapy: Molecular mechanisms of action. Eur. J. Pharmacol..

[8] Owen D.H., Halmos B., Puri S., Qin A., Ismaila N., Abu Rous F., Alluri K., Freeman-Daily J., Malhotra N., Marrone K.A. (2025). Therapy for Stage IV Non-Small Cell Lung Cancer Without Driver Alterations: ASCO Living Guideline, Version 2025.1. J. Clin. Oncol..

[9] Topalian S.L., Hodi F.S., Brahmer J.R., Gettinger S.N., Smith D.C., McDermott D.F., Powderly J.D., Carvajal R.D., Sosman J.A., Atkins M.B. (2012). Safety, activity, and immune correlates of anti-PD-1 antibody in cancer. N. Engl. J. Med..

[10] Fournel L., Wu Z., Stadler N., Damotte D., Lococo F., Boulle G., Segal-Bendirdjian E., Bobbio A., Icard P., Tredaniel J. (2019). Cisplatin increases PD-L1 expression and optimizes immune check-point blockade in non-small cell lung cancer. Cancer Lett..

[11] Peng J., Hamanishi J., Matsumura N., Abiko K., Murat K., Baba T., Yamaguchi K., Horikawa N., Hosoe Y., Murphy S.K. (2015). Chemotherapy Induces Programmed Cell Death-Ligand 1 Overexpression via the Nuclear Factor-kappaB to Foster an Immunosuppressive Tumor Microenvironment in Ovarian Cancer. Cancer Res..

[12] Garassino M.C., Gadgeel S., Speranza G., Felip E., Esteban E., Domine M., Hochmair M.J., Powell S.F., Bischoff H.G., Peled N. (2023). Pembrolizumab Plus Pemetrexed and Platinum in Nonsquamous Non-Small-Cell Lung Cancer: 5-Year Outcomes From the Phase 3 KEYNOTE-189 Study. J. Clin. Oncol..

[13] Gandhi L., Rodriguez-Abreu D., Gadgeel S., Esteban E., Felip E., De Angelis F., Domine M., Clingan P., Hochmair M.J., Powell S.F. (2018). Pembrolizumab plus Chemotherapy in Metastatic Non-Small-Cell Lung Cancer. N. Engl. J. Med..

[14] Wei H., Chen Q., Lin L., Sha C., Li T., Liu Y., Yin X., Xu Y., Chen L., Gao W. (2021). Regulation of exosome production and cargo sorting. Int. J. Biol. Sci..

[15] Batista B.S., Eng W.S., Pilobello K.T., Hendricks-Munoz K.D., Mahal L.K. (2011). Identification of a conserved glycan signature for microvesicles. J. Proteome Res..

[16] Johnstone R.M., Adam M., Hammond J.R., Orr L., Turbide C. (1987). Vesicle formation during reticulocyte maturation. Association of plasma membrane activities with released vesicles (exosomes). J. Biol. Chem..

[17] Sengupta P., Dutta S., Jallo M.K., Rosas I.M., Roychoudhury S. (2026). Seminal Plasma and Extracellular Vesicles as Molecular Gatekeepers: Oxidative Stress, Endocrine Crosstalk, and Biomarker Discovery in Male Infertility. Curr. Issues Mol. Biol..

[18] Magni F., Van Der Burgt Y.E., Chinello C., Mainini V., Gianazza E., Squeo V., Deelder A.M., Kienle M.G. (2010). Biomarkers discovery by peptide and protein profiling in biological fluids based on functionalized magnetic beads purification and mass spectrometry. Blood Transfus..

[19] Li Z., Fang R., Fang J., He S., Liu T. (2018). Functional implications of Rab27 GTPases in Cancer. Cell Commun. Signal.

[20] Li W., Hu Y., Jiang T., Han Y., Han G., Chen J., Li X. (2014). Rab27A regulates exosome secretion from lung adenocarcinoma cells A549: Involvement of EPI64. APMIS.

[21] Koh H.M., Song D.H. (2019). Prognostic role of Rab27A and Rab27B expression in patients with non-small cell lung carcinoma. Thorac. Cancer.

[22] Postow M.A., Callahan M.K., Wolchok J.D. (2015). Immune Checkpoint Blockade in Cancer Therapy. J. Clin. Oncol..

[23] Hodi F.S., Chiarion-Sileni V., Gonzalez R., Grob J.J., Rutkowski P., Cowey C.L., Lao C.D., Schadendorf D., Wagstaff J., Dummer R. (2018). Nivolumab plus ipilimumab or nivolumab alone versus ipilimumab alone in advanced melanoma (CheckMate 067): 4-year outcomes of a multicentre, randomised, phase 3 trial. Lancet Oncol..

[24] Pacheco J.M., Camidge D.R., Doebele R.C., Schenk E. (2019). A Changing of the Guard: Immune Checkpoint Inhibitors With and Without Chemotherapy as First Line Treatment for Metastatic Non-small Cell Lung Cancer. Front. Oncol..

[25] Chen G., Huang A.C., Zhang W., Zhang G., Wu M., Xu W., Yu Z., Yang J., Wang B., Sun H. (2018). Exosomal PD-L1 contributes to immunosuppression and is associated with anti-PD-1 response. Nature.

[26] Wang J., Guo W., Wang X., Tang X., Sun X., Ren T. (2023). Circulating Exosomal PD-L1 at Initial Diagnosis Predicts Outcome and Survival of Patients with Osteosarcoma. Clin. Cancer Res..

[27] Li S., Yi M., Dong B., Jiao Y., Luo S., Wu K. (2020). The roles of exosomes in cancer drug resistance and its therapeutic application. Clin. Transl. Med..

[28] Poggio M., Hu T., Pai C.C., Chu B., Belair C.D., Chang A., Montabana E., Lang U.E., Fu Q., Fong L. (2019). Suppression of Exosomal PD-L1 Induces Systemic Anti-tumor Immunity and Memory. Cell.

[29] Lee C.H., Bae J.H., Choe E.J., Park J.M., Park S.S., Cho H.J., Song B.J., Baek M.C. (2022). Macitentan improves antitumor immune responses by inhibiting the secretion of tumor-derived extracellular vesicle PD-L1. Theranostics.

[30] de Miguel-Perez D., Russo A., Arrieta O., Ak M., Barron F., Gunasekaran M., Mamindla P., Lara-Mejia L., Peterson C.B., Er M.E. (2022). Extracellular vesicle PD-L1 dynamics predict durable response to immune-checkpoint inhibitors and survival in patients with non-small cell lung cancer. J. Exp. Clin. Cancer Res..

[31] Jia T., Zhang Q., Xu H., Liu H., Gu X. (2023). The function of miR-637 in non-small cell lung cancer progression and prognosis. Pulmonology.

[32] Li J., Wu D.M., Han R., Yu Y., Deng S.H., Liu T., Zhang T., Xu Y. (2020). Low-Dose Radiation Promotes Invasion and Migration of A549 Cells by Activating the CXCL1/NF-kappaB Signaling Pathway. Onco Targets Ther..

[33] Chen H., Liu L., Xing G., Zhang D., Niumuqie A., Huang J., Li Y., Zhao G., Liu M. (2025). Exosome tropism and various pathways in lung cancer metastasis. Front. Immunol..

[34] Peng S., Wang R., Zhang X., Ma Y., Zhong L., Li K., Nishiyama A., Arai S., Yano S., Wang W. (2019). EGFR-TKI resistance promotes immune escape in lung cancer via increased PD-L1 expression. Mol. Cancer.

[35] Chen Y., Song W., Gao Y., Dong X., Ji X. (2022). Increased PD-L1 Expression in Acquired Cisplatin-Resistant Lung Cancer Cells via Mir-181a. Tohoku J. Exp. Med..

[36] Qin X., Yu S., Zhou L., Shi M., Hu Y., Xu X., Shen B., Liu S., Yan D., Feng J. (2017). Cisplatin-resistant lung cancer cell-derived exosomes increase cisplatin resistance of recipient cells in exosomal miR-100-5p-dependent manner. Int. J. Nanomed..

[37] Irep N., Inci K., Tokgun P.E., Tokgun O. (2024). Exosome inhibition improves response to first-line therapy in small cell lung cancer. J. Cell Mol. Med..

[38] Li X.Q., Liu J.T., Fan L.L., Liu Y., Cheng L., Wang F., Yu H.Q., Gao J., Wei W., Wang H. (2016). Exosomes derived from gefitinib-treated EGFR-mutant lung cancer cells alter cisplatin sensitivity via up-regulating autophagy. Oncotarget.

[39] Trajkovic K., Hsu C., Chiantia S., Rajendran L., Wenzel D., Wieland F., Schwille P., Brugger B., Simons M. (2008). Ceramide triggers budding of exosome vesicles into multivesicular endosomes. Science.

[40] Kosaka N., Iguchi H., Yoshioka Y., Takeshita F., Matsuki Y., Ochiya T. (2010). Secretory mechanisms and intercellular transfer of microRNAs in living cells. J. Biol. Chem..

[41] Meneses K.M., Pandya P., Lindemann J.A., Al-Qasrawi D.S., Argo R.A., Weems C.M., Beetler D.J., Vijay G.V., Yan I.K., Wolfram J. (2023). RAB27B Drives a Cancer Stem Cell Phenotype in NSCLC Cells Through Enhanced Extracellular Vesicle Secretion. Cancer Res. Commun..

[42] Li C., Li C., Zhi C., Liang W., Wang X., Chen X., Lv T., Shen Q., Song Y., Lin D. (2019). Clinical significance of PD-L1 expression in serum-derived exosomes in NSCLC patients. J. Transl. Med..

[43] Li D., Meng D., Niu R. (2020). Exosome-Reversed Chemoresistance to Cisplatin in Non-Small Lung Cancer Through Transferring miR-613. Cancer Manag. Res..

